# Added value of hyperdense lumen sign in prediction of acute central and peripheral pulmonary embolism on non-contrast CT chest

**DOI:** 10.1186/s43055-021-00462-9

**Published:** 2021-03-25

**Authors:** Hend Galal Eldeen Mohamed Ali Hassan, Nivan Hany Khater, Remon Zaher Elia

**Affiliations:** 1 Radiodiagnosis Department, Faculty of Medicine, Ain shams University, Cairo, Egypt; 2 Radiodiagnosis Department, Faculty of Medicine, Ain shams University, Cairo, Egypt

**Keywords:** Pulmonary embolism, Non-contrast CT chest, Hyperdense lumen sign

## Abstract

**Background:**

Pulmonary embolism (PE) is a common condition with considerable morbidity and mortality; it is more often diagnosed post-mortem by pathologists than in vivo by clinicians. Prompt and accurate diagnosis is difficult because PE may be clinically silent, the symptoms are vague and nonspecific, and in addition, there is no definitive, non-invasive diagnostic test to establish its diagnosis. The aim of this study is to assess the reliability of detection of acute central and peripheral pulmonary embolism (PE) on non-contrast CT especially when no possible alternative is available as in allergic cases or emergency, patients with history of renal disease, or in cases where PE is not the leading diagnosis. CT pulmonary angiography study served as our gold standard.

**Results:**

Eighty adult patients were included in our study; 44 were females and 36 males most of which were complaining of dyspnea and chest pain. Acute central pulmonary embolism was confirmed by CTPA. They all underwent a pre-contrast study just prior to the CTPA. Presence of high attenuation emboli in any of the main pulmonary vessels was our key for diagnosis of acute embolism. Non-contrast CT chest diagnosed 26 of the 47 cases confirmed by CTPA. The hyperdense lumen sign had an overall sensitivity of 55.3%, specificity of 100%, positive predictive value (PPV) of 100%, and negative predictive value of 61.1%. The accuracy of non-contrast CT chest study was evaluated using CTPA as our gold standard.

**Conclusion:**

Non-contrast CT chest is a good indicator in predicting central and peripheral pulmonary embolism, particularly in cases of emergency, those unable to take intravenous contrast for angiography, or in cases where pulmonary embolism is not the leading diagnosis.

## Background

Pulmonary embolism is a potentially fatal disease of which the clinical presentation may be silent [1]. In a good number of cases, it may even pass undiagnosed as autopsy series suggest that the true number is around threefold higher, with a number of patients dying without the diagnosis ever even made [2]. Based on the extent of emboli, the fatality rate may reach up to 30% if left untreated [3].

In around 97% of patients, the presenting symptoms include dyspnea, chest pain, or tachypnea with no history of pulmonary or cardiac disease [4]. The diagnosis of PE cannot be excluded solely through clinical evaluation or suspicion, thus requiring further investigations [5]. In many institutions, CTPA is an initial established imaging modality for pulmonary embolism diagnosis [6]. Other tools for diagnosis are ventilation-perfusion (VQ) nuclear medicine imaging with echocardiography and lower limb venous duplex required in a selected group of patients [7].

However, in a number of situations, a non-contrast CT chest may be the only imaging modality possible such as in patients with allergies to iodinated contrast or those with known history of renal insufficiency [8]. Central pulmonary embolism also results in severe hemodynamic changes requiring timely intervention and waiting for the renal function lab tests may delay the diagnosis, thus the necessity of an alternative fast method as a non-contrast CT study [9].

A limited number of studies have focused on the utility of non-contrast CT chest in PE detection based on the diagnosis of high attenuation thrombi in the pulmonary vessels [10].

Furthermore, including an unenhanced study as part of the CTPA protocol is useful for a number of other reasons as evaluation of the lung parenchyma and chest wall and for identification of any calcified lesions [11].

The aim of this study is to assess the validity and reliability of non-contrast CT chest study in detecting central pulmonary thromboembolism in comparison to CTPA as regards its sensitivity and specificity.

## Methods

### Patients

Our 6-month prospective study was performed from May 2020 till October 2020. Eighty adult patients were included in this study of which 44 were females and 36 males with a median age of 52 and a mean 54.18 with a range of 35–72 years. Clinical presentation ranged from chest pain (50 patients), dyspnea (48 patients), hemoptysis (14 patients), tachypnea (19 patients), and tachycardia (22 patients). All were subjected to proper history taking. A written consent was taken from all the patients according to the rules of our ethical committee.

#### Exclusion criteria

- Patients not suitable for intravenous injection of contrast media (impaired renal functions or known allergy to contrast media)
- Pregnant females

### Technique of examination

All patients were examined at the CT unit of Radiology Department using GE OPTIMA 66SE MSCT 64 CT scanner.

All patients were told to fast for 6 h before the examination. Twenty-Gauge IV cannula was placed in an antecubital vein, the procedure was explained to the patient, and metallic objects were removed. Patient was placed supine, headfirst on the CT table, and instructed not to move during the scan.

First, a pre-contrast CT imaging of the chest with limited field of view (FOV) only to pulmonary region was taken followed by immediately contrast-enhanced chest CT scan in highly suspicious cases, or extend the non-contrast CT FOV to cover whole chest to exclude any other possibility of chest pain or COVID-19 infection and postponed CTPA in mildly suspicious cases to avoid radiation exposure, in which a dual-head automated injector was connected to the antecubital vein cannula with a scanogram collimation used 0.75mm with rotation time of 0.37–0.42 s, where axial cuts were obtained from suprasternal level till below the diaphragm with additional sagittal and coronal reconstruction images.

Monophasic injection of average 80 ml non-ionic low osmolar contrast material (LOCM), e.g., ultravist 370, is preferred for better vascular contrast density and safety for borderline creatinine level (1.2 ml/kg body weight) at a flow rate of 4 ml**/**s. Twenty milliliters of normal saline was injected at the same rate before and after contrast injection to check the IV line for extravasation and as a wash out of the bolus respectively. The scanning delay was determined using the bolus tracking technique in the lumen of the pulmonary trunk. The x-ray tube voltage setting was 120 kV, and the current average was 200 mA, depending on patient size and the heat limitations of the tube. Transverse sections were reconstructed on a workstation with a section width of 1.25 mm at an interval of 0.7mm (0.65 mm overlap), resulting in a mean of 583 transverse images (range, 531–632 images).

Postprocessing using maximum intensity projection (MIP), volume rendering, and shaded surface display was done. MIP images were created in both coronal and oblique planes, in conjunction with axial data to allow maximum vessels visualization.

### Statistical analysis of data

Gold standard for the diagnosis of PE was CTPA. Recorded data were analyzed using Statistical Package for Social Science (IBM Corp, released 2013. IBM SPSS statistics for windows, V. 22.0. Armonk, NY, USA). Parametric quantitative data were expressed as mean± standard deviation (SD). Non-parametric data were expressed as median with inter-quartile range (IQR). Qualitative data were described as frequency and percentage.

The cross tabulation was used to evaluate the diagnostic performance of non-contrast CT for diagnosing central pulmonary embolism as compared to the contrast enhanced diagnosis with calculation of sensitivity, specificity, and accuracy. Sensitivity is the capacity of the test to correctly identify diseased individuals, specificity is the capacity of the test to correctly exclude individuals who are free of the disease, and accuracy is rate of agreement (true positives + true negatives)/total tested × 100). The Pearson chi-square test was used to compare the binary categorical variable. Cohen’s kappa coefficient (*K*) was performed to assess the inter-method agreement. Kappa agreement was interpreted as 0.01–0.20, slight agreement; 0.21–0.40, fair agreement; 0.41–0.60, moderate agreement; 0.61–0.80, substantial agreement; and 0.81–0.99, almost perfect agreement. *P* value ˂0.05 was considered significant. *P* value ˃0.05 was considered insignificant.

## Results

Eighty adult patients were included in our study (44 females, 36 males). Ages ranged from 35 to 72 years with a median of 52 years and a mean of 54.18 years. There was no statistically significant difference between the mean age groups of those with (mean 55.77±1.46 SD) and without (mean 51.91±1.24 SD) PE. A total of 47/80 patients were diagnosed with PE as confirmed by CTPA with male predominance 53% and 33/80 negative of PE with female predominance 66%.

Most common presenting symptom was chest pain (50/80 patients) and dyspnea (48/80 patients). Other associated indirect signs seen on the non-contrast CT highest were pleural effusion (26/80 cases), followed by peripheral wedge-shaped opacity (12/80 cases) and lastly pulmonary artery dilatation (9/80 cases).

CTPA showed positive thromboembolism in 47/80 cases. Highest incidence of acute pulmonary embolism involved both pulmonary arteries in 15/47 cases, followed by the right main pulmonary artery in 15/47 cases, the left main pulmonary artery in 13/47 of the cases, and 4/47 cases involving subsegmental peripheral pulmonary branch (Table 1).

**Table 1:** Distribution of site of thrombus in pulmonary artery among positive cases by CTPA

CTPA		Frequency	Percent
**Negative**	No thrombus	33	100.0
**Positive**	Right main	15	31.9
	Left main	13	27.7
	Bilateral	15	31.9
	Peripheral	4	8.5
	Total	47	100.0

The number of cases found to be positive for acute pulmonary embolism on non-contrast CT chest were 26/80 cases representing around 32.5% of all the examined patients and 55.3% among CTPA proven positive cases with 100% accuracy in excluding PE as CTPA (Table 2) shows the efficacy of non-contrast CT chest in detecting high attenuation thrombi as compared to CTPA.

**Table 2:** Non-contrast CT significance and accuracy percentage in comparison to gold standard CTPA-positive cases

Non contrast CT finding/positive CTPA		Frequency	Percent
Negative	Negative	33	100.0
Positive	Negative	21	44.7
	Positive	26	55.3
	Total	47	100.0

Table 3 shows the diagnostic accuracy of non-contrast CT chest in detection of central pulmonary embolism in this study reaching to 73.7%.

**Table 3:** Diagnostic accuracy of non-contrast CT chest in detection of central pulmonary embolism in our study

Parameter	TP	TN	FP	FN	Accuracy	Sensitivity	Specificity	PPV	NPV
**Non contrast CT**	26	33	0	21	73.75%	55.3%	100.0%	100.0%	61.1%

*TP* total positive, *TN* total negative, *FP* false positive, *FN* false negative, *PPV* positive predictive factor, *NPV* negative predictor factor

## Discussion

Pulmonary embolism is a potentially fatal condition and its diagnosis is a challenging task, both clinically and radiologically [12]. Clinical assessment of the patient is the first and most crucial step to reduce unnecessary imaging which has undesirable consequences as increased cost and ionizing radiation exposure [13]. However, in a good number of cases, clinical diagnosis may be problematic as symptoms may range from silent to hemodynamic instability [14]. Thus, the need for a ready available modality for timely diagnosis.

The introduction of the relatively non-invasive spiral CT angiography has shown to reliably exclude clinically important PE [15]. The development of multi-detector CT has led to improved visualization of peripheral pulmonary arteries and small sub-segmental emboli [16]. However in patients with allergies to iodinated contrast material or with elevated serum creatinine levels and those patients with nonspecific cardiopulmonary signs and symptoms, non-contrast CT chest may be the only ready accessible modality.

It is important to be aware of the hyperdense thrombus to help diagnose acute PE in patients undergoing non-contrast CT of the chest. Visualization of the clot is likely related to the age of the clot with increased density in the vessel either due to direct visualization of the thrombus itself or as a result of local slow intravascular blood flow due to intra-arterial thrombi [17].

This study included 80 adult patients clinically suspicious of PE. Age of the patients ranged from 35 to 72 years old with a mean age 54.18 years. Majority of our cases were females representing 44 patients (55% of the cases). A study done by Venkatesh et al. [18] also showed predominant female patients with 60%, 57%, and 67% incidence.

Symptoms of PE are typically sudden in onset and include dyspnea, tachypnea, chest pain of a “pleuritic” nature (worsened by breathing), cough, and hemoptysis.

The clinical presentation of patients was as follows: 50 patients presented by chest pain representing 62.5%, 48 patients had dyspnea representing 60%, 19 patients had tachypnea representing 23.8%, 22 patients presented by tachycardia representing 27.5%, and 14 patients presented by hemoptysis representing 17.5 %. Our study agreed with Tambe et al. [19] that showed that the most common clinical symptoms were sudden and/or unexplained chest pain, dyspnea, malaise, syncope, or shortness of breath. Another study by Crichlow et al. [20] showed that the most common presenting signs and symptoms were shortness of breath (77%), followed by chest pain (74.3%).

From the 80 patients, 47 were proved positive by CTPA. CTPA was the gold standard in our study. Visualization of complete or partial intraluminal filling defects surrounded by the contrast-enhanced blood pool in the central and subsegmental pulmonary arteries is a direct sign of PE. Distribution of the true positive cases of pulmonary thrombosis among included patients was as follows: 15 patients showed a thrombus within both main pulmonary arteries (18.7%), 15 patients showed a thrombus in the right main pulmonary artery (18.7%), 13 patients had a thrombus in the left main pulmonary artery (16.2%), and last 4 patients had thrombus in subsegmental peripheral pulmonary branch (5%).

**Fig. 1:**
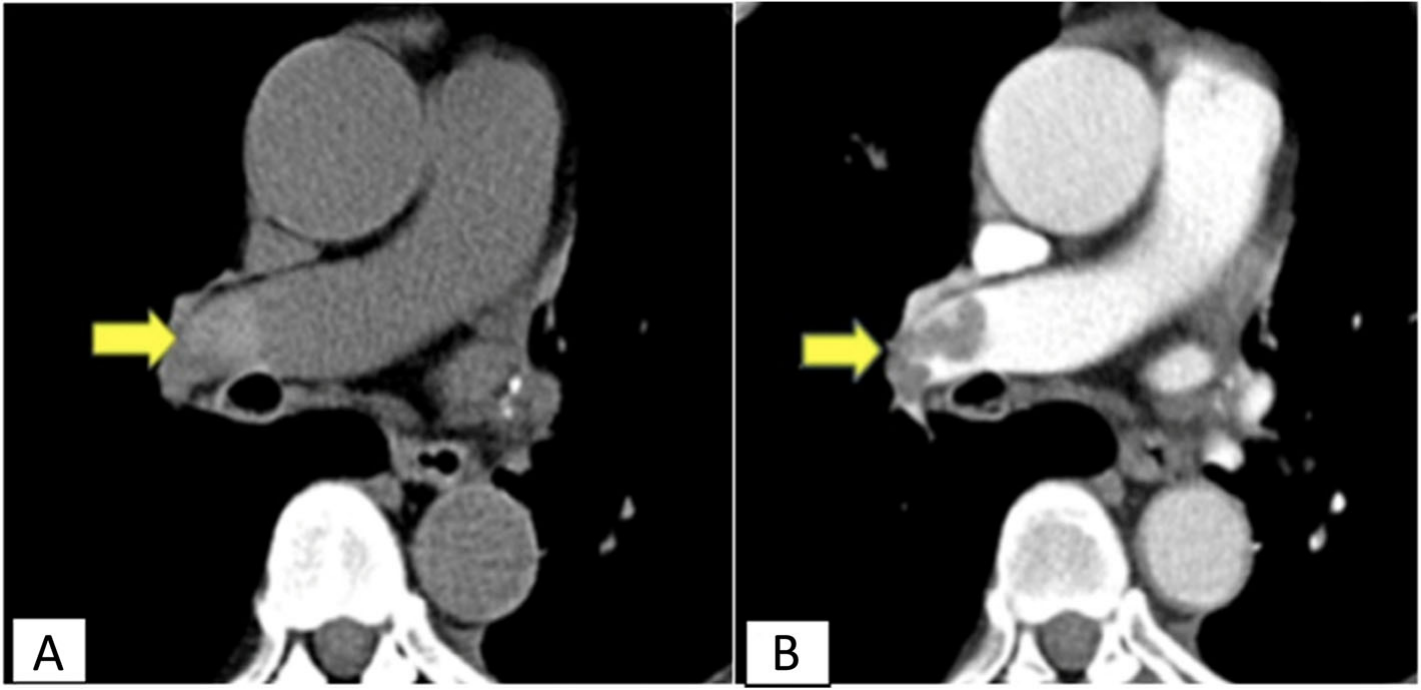
**a** Axial non contrast CT image. **b** Axial CTPA image shows a case of acute pulmonary embolism with hyperdense thrombus seen at right main pulmonary artery (arrows in **a**) with corresponding filling defect at right main pulmonary artery (arrows in **b**)

**Fig. 2:**
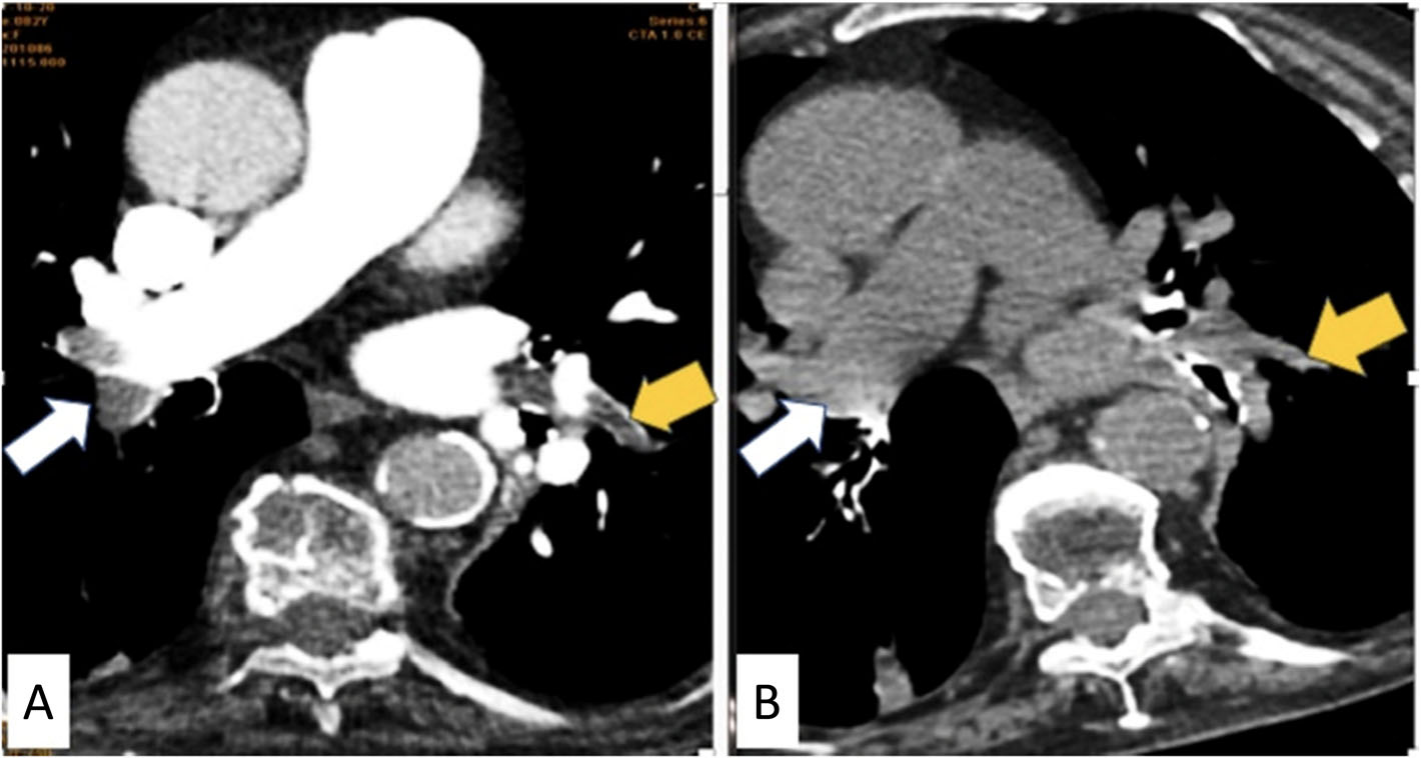
**a** Axial CTPA image. **b** Axial non-contrast image shows a case of acute pulmonary embolism with hyperdense thrombus seen at right distal main pulmonary artery (white arrows).and left lower sub-segmental peripheral branch (yellow arrows) with corresponding filling defect at CTPA

Out of the 47 CTPA proved positive patients, 26 cases were positive by non-contrast CT chest depending on hyper dense lumen sign either in central main pulmonary branch or subsegmental peripheral branches (Figs. 1 and 2), with an overall sensitivity of 55.3%, specificity of 100% positive predictive value of 100%, and negative predictive value of 61.1% in the detection of emboli located within the main pulmonary arteries (central emboli). And 21 cases were false positive by non-contrast CT chest attributing to sluggish blood flow mimicking hyperdense sign; as regards false-negative cases, it is attributed to small sized embolus. There was a moderate degree of agreement according to Kappa method (0.505) with *p* value 0.000. However, chi-square detection rate of central pulmonary embolism for CTPA was significantly higher than that of non-contrast CT with a *p* value of 0.000.

Tatco and Piedad [21] reported an overall sensitivity of only 36% for detecting central PE, which is significantly lower than our study. On the other hand, Cobelli et al. [22] reported a 41.2% sensitivity and Kanne et al. [23] found that 46.1% of their unenhanced scans were positive for PE.

**Fig. 3:**
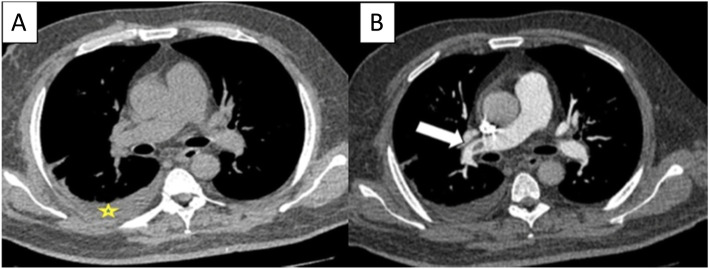
**a** Axial non contrast CT image. **b** Axial CTPA image shows a case of false-negative finding of pulmonary embolism in non-contrast CT chest associated with right pleural effusion (asterisk in **a**), with positive finding in CTPA as hypo dense filling defect seen partially occluding right main pulmonary artery (arrows in **b**)

A number of indirect findings were also seen on the non-contrast CT chest including pleural effusion which was the most common finding seen in 26 patients (32.5%) of all cases (Fig. 3). Second most common finding was a peripheral wedge-shaped opacity in 12 patients (15%) of all cases, followed by pulmonary artery dilatation in 9 cases (11.3%) of all cases. A study done by Pfeil et al. [24] reported that wedge-shaped opacity was the most frequent indirect sign.

During the pandemic of COVID-19 which occurred at the same time of this study, the non-contrast CT was done to exclude viral infection followed by contrast CT after 48 h to exclude pulmonary embolism, in which retrospectively reviewing non-contrast CT revealing presence of hyperdense lumen sign, by which the importance of including this sign in reporting non-contrast CT is highlighted.

There were some limitations in this study; mainly, number of cases is still limited which hindered the possibility to study the usefulness of the hyperdense lumen sign in the segmental or subsegmental branches. Factors like motion artifacts, partial volume averaging, and image noise which almost always affect segmental and more peripheral pulmonary arteries are some of the possible causes why the hyper dense lumen sign is less useful in detecting peripherally located thrombi. Factors that may affect the visualization of a clot, such as the age of the clot, the patient’s hematocrit level at the time of imaging, and probably the patient’s hematocrit level at the time of formation of the clot in the venous system were not considered in this study.

## Conclusion

The utility of unenhanced CT chest has been addressed in only a few studies and its recognition may be useful in acute pulmonary embolism, especially when not clinically suspected. Despite CTPA being the gold standard study and much more sensitive, non-contrast CT has a good role in detecting central pulmonary embolism in those patients not able to perform CTPA as in allergic or renal insufficiency cases or in those with non-specific cardiopulmonary symptoms.

In our study, non-contrast chest CT scans has a good role in evaluation of PE through detection the hyper dense lumen sign that is a good indicator of acute pulmonary thromboembolism, particularly in cases involving the central pulmonary arteries.

## Data Availability

The datasets used and/or analyzed during the current study are available from the corresponding author on reasonable request**.**

## Abbreviations

PE: Pulmonary embolism
CT: Computed tomography
CTPA: CT pulmonary angiography
COVID: Coronavirus disease
FOV: Field of view
MIP: Maximum intensity projection
VR: Volume rendering
